# Low-loss hybrid germanium-on-zinc selenide waveguides in the longwave infrared

**DOI:** 10.1515/nanoph-2023-0698

**Published:** 2024-01-08

**Authors:** Dingding Ren, Chao Dong, Jens Høvik, Md Istiak Khan, Astrid Aksnes, Bjørn-Ove Fimland, David Burghoff

**Affiliations:** Department of Electrical Engineering, University of Notre Dame, Notre Dame, USA; Department of Electrical and Computer Engineering, University of Texas at Austin, Austin, USA; Department of Electronic Systems, Norwegian University of Science and Technology (NTNU), Trondheim, Norway

**Keywords:** longwave infrared, germanium, zinc selenide, integrated photonics, quantum cascade laser

## Abstract

The longwave infrared (LWIR) range, which spans from 6 µm to 14 µm, is appealing for sensing due to strong molecular fingerprints in this range. However, the limited availability of low-loss materials that can provide higher-index waveguiding and lower-index cladding in the LWIR range presents challenges for integrated photonics. In this work, we introduce a low-loss germanium-on-zinc selenide (GOZ) platform that could serve as a versatile platform for nanophotonics in the LWIR. By bonding high-quality thin-film germanium (Ge) to a zinc selenide (ZnSe) substrate, we demonstrate transparency from 2 µm to 14 µm and optical losses of just 1 cm^−1^ at 7.8 µm. Our results demonstrate that hybrid photonic platforms could be invaluable for overcoming the losses of epitaxially grown materials and could enable a wide range of future quantum and nonlinear photonics.

## 1 Introduction

The LWIR portion of the spectrum, which spans from 6 µm to 14 µm, holds considerable promise for integrated photonic sensors in areas such as environmental monitoring, early disease detection, and production quality control [1], [2], [3]. The value of the LWIR spectrum is rooted in its resonant interactions with the rovibrational transitions of molecules, which result in enhanced sensitivity and selectivity in molecular sensing. For instance, amides exhibit a distinct absorption around 6 µm, aiding in the detection of colitis [4] and the analysis of various protein structures [5] by examining the amides ratio in the LWIR spectrum. At longer wavelengths, interactions with heavier molecules become more pronounced due to their reduced resonance frequencies. Additionally, LWIR light could outperform mid-wave infrared, short-wave infrared, and visible light in adverse weather conditions, thanks to reduced scattering at these longer wavelengths [6], [7], making it potentially valuable in LIDAR applications.

However, the progression of integrated LWIR photonics remains limited by the availability of low-loss materials. There are many suitable photonics platforms at shorter wavelengths, perhaps most famously Si_3_N_4_ and silicon-on-insulator (SOI), but all of these are incompatible with longer-wavelength operation. Most oxides and nitrides are extremely lossy – for example, Si_3_N_4_ is absorptive beyond 4 µm [8] – and even silicon (Si) has high losses beyond 7 µm due to multiphonon absorption [9]. Crystalline fluorides have exhibited promise in low-loss mid-infrared devices [10], [11] and have demonstrated comb generation at wavelengths extending above 4 µm [12], but doing this in a fully integrated platform is challenging. III–Vs are also highly appealing due to their natural compatibility with sources like quantum cascade lasers [13], [14] but have higher losses toward the longer wavelength side of the LWIR. As a result, many of the interesting nanophotonic devices that have been created at shorter wavelengths, like frequency combs [15], [16] and integrated nonlinear optics, have relied on active quantum cascade media to overcome the losses at longer wavelengths. This strategy limits the optical bandwidths that can be achieved and produces a significant amount of spontaneous emission, which could possibly have a negative impact for emerging quantum technologies (e.g., quantum combs [17], quantum frequency conversion [18], quantum sensing [19], etc.).

Ge boasts a higher atomic weight than Si, which reduces the multiphonon absorption frequency [20]. Thus, Ge is a more promising low-loss material than Si for LWIR applications, encompassing the entire second atmospheric transparency window [21]–[25]. Furthermore, Ge’s atomic structure closely resembles Si’s, allowing compatibility with many standardized Si fabrication processes. Recently, significant progress has been made in the epitaxial growth of Si-containing Ge photonic platforms. For example, graded-index SiGe resonator have shown Q factors of 10^5^ from 7.5 µm to 9 µm [26], and SiGe-on-Si ring resonator has demonstrated quality factors of 2.36 × 10^5^ at 4.18 µm [27]. Still, any incorporation of Si in the epitaxy blueshifts the multiphonon absorption, narrowing the transparency window. Thus, the optimal Ge-based photonics platform would incorporate native Ge and no Si.

In previous work, it was shown that native Ge waveguides could be integrated onto glass substrates in order to couple light into the high-Q suspended Ge whispering gallery mode microresonators [28]. However, SiO_2_ glass has optical losses exceeding 10,000 cm^−1^ in the LWIR [29]. Although Ge possesses a substantial refractive index of 4 in the LWIR and losses as low as 0.5 dB cm^−1^ in the LWIR, the evanescent optical field in the cladding induces significant loss, resulting in a waveguide loss of approximately 39 dB cm^−1^. This rendered it unsuitable for integrated photonics applications. To approach the low losses of native Ge, one must substitute glass with a low-loss material in the LWIR [30]. Additionally, this material should have a lower refractive index than Ge to serve as an effective cladding material. Zinc selenide (ZnSe), a II–VI material composed of heavy elements with a refractive index of about 2.4 in the LWIR, is a logical choice due to its exceptionally low-onset frequency for multiphonon absorption and remarkably low losses in the LWIR [31], [32], [33]. It is for this reason that it is commonly used as an infrared material, for example, as the window material for high-power infrared CO_2_ lasers. Importantly, the thermal expansion of ZnSe matches reasonably well with Ge, making it compatible with direct wafer bonding.

In this work, we use heterogeneous integration to create the first GOZ waveguides. By combining direct bonding and mechanical thinning processes, we are able to realize low-loss native Ge thin films on ZnSe that can be nanofabricated into waveguides. Optical transmission measurements were conducted using a commercial continuously tunable distributed feedback quantum cascade laser (DFB-QCL) and a broadband infrared glowbar, and our results demonstrate that this platform has broadband transparency (covering 2–14 µm) and covers the entire LWIR spectrum with optical losses as low as 1 cm^−1^ at 7.8 µm.

## 2 Fabrication method

**Figure 1:**
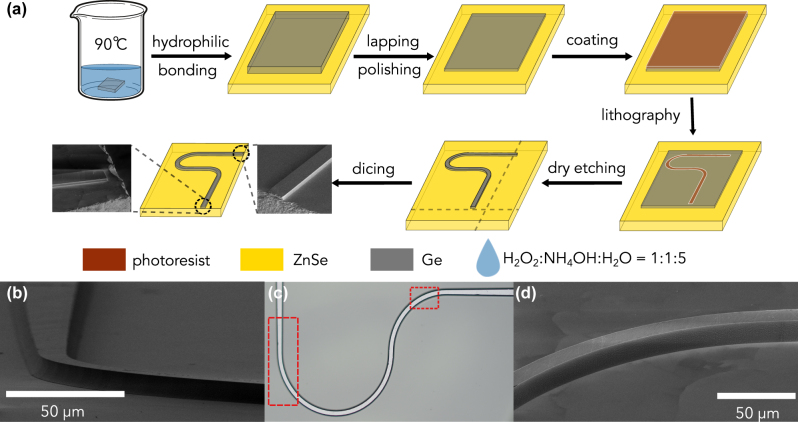
Fabrication of the GOZ waveguide. (a) Schematic representation of the fabrication steps, with insets of SEM images showing both the front (dicing) and back (dry etching) facets of the waveguide. The dashed gray line indicates the dicing cut location. (b) Electron microscopy image showcasing the smooth transition from the Ge waveguide to the ZnSe substrate, free of defects. (c) Optical microscope image of the waveguide, with the region of interest demarcated by red-dashed rectangles on the left and right for (b) and (d), respectively. (d) A tilted perspective reveals the waveguide’s smooth curvature and the visible ZnSe polycrystalline grain boundaries on the substrate, highlighting a well-bonded Ge-ZnSe interface.

Figure 1a presents a schematic overview of the fabrication process. The creation of the fully integrated Ge-on-ZnSe waveguide began with a hydrophilic wafer bonding technique, mirroring the process we previously developed for the suspended Ge whispering gallery mode microresonator [28]. To counteract surface degradation from oxidation, both Ge and ZnSe were briefly immersed in a hot bath of H_2_O_2_:NH_4_OH:H_2_O (with a volume ratio of 1:1:5) at 90 °C for 5 s. This was succeeded by a compressed annealing process using a force of 3000 N at 300 °C for 60 min. Postpolishing, the Ge thin film on the ZnSe substrate measured approximately 5 µm in thickness. The atomic force microscopy analysis indicated that the surface roughness of the polished Ge is approximately 10 nm, characterized by sporadic spike-like features not exceeding 60 nm in height [28]. A 90° curved waveguide was subsequently delineated using a maskless laser writer, followed by an ICP-RIE dry etching process utilizing SF_6_ chemistry. To inspect the interface between the Ge waveguide and the ZnSe substrate, the substrate was tilted to be nearly parallel to the incident electron beam’s direction. As shown in Figure 1b, a seamless transition from Ge to ZnSe was observed, devoid of defects. This waveguide region is highlighted by a red-dashed rectangle on the left side in the optical microscope image in Figure 1c. Tilting the substrate to a steeper angle relative to the incident electron beam revealed the waveguide’s smooth curvature and the ZnSe polycrystalline grain boundaries on the substrate, as depicted in Figure 1d. These observations confirm a well-bonded interface between Ge and ZnSe, suggesting that GOZ can be effectively integrated into LWIR photonics.

## 3 High-resolution transmission measurement

To evaluate the Ge waveguide’s performance in LWIR light coupling, the front-end facet (for light coupling) was formed by dicing, aiming to align closely with the ZnSe substrate's end facet, as depicted in the right inset of Figure 1a. The back-end facet (for signal collection) was created through dry etching as shown in the left inset of Figure 1a. Our optical setup involved a series of off-axis parabolas (OAPs) to expand the QCL beam to 30 mm in diameter, followed by a one-inch diameter OAP with a one-inch focal length for focusing onto the waveguide’s end facet. The emitted signal was collected using a two-inch diameter OAP with a four-inch focal length. The beam diameter was reduced to 1 inch before entering the detector through a ZnSe lens. Two pinholes at the confocal planes of the OAP mirror pairs were used to eliminate interference from scattered laser signals. The QCL wavelength scan was controlled by simultaneously adjusting the current and temperature. For further details on the optical measurement setup using our commercial DFB-QCL, readers are referred to our previous work [28].

**Figure 2:**
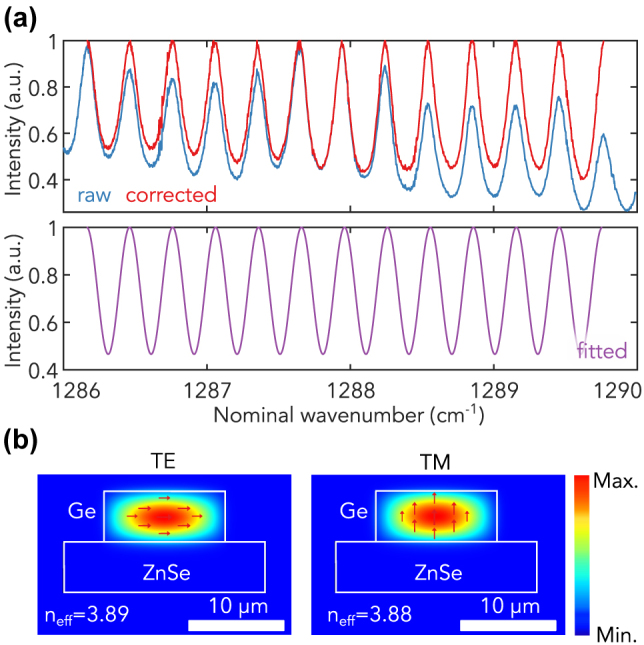
Characterization of the GOZ waveguide. (a) Transmitted signal from the Ge waveguide when the DFB-QCL laser wavelength was scanned from 1286 cm^−1^ to 1290 cm^−1^. A clear resonance is observed. (b) COMSOL simulation illustrating the confined mode inside Ge waveguide on the ZnSe substrate.

A laser output in TE mode of approximately 5 mW from the DFB-QCL, which has a maximum optical power of 250 mW, was directed onto the end facet of the Ge waveguide. The light that emerged from the waveguide was collected by an OAP mirror and subsequently detected using a MgCdTe (MCT) detector. As depicted by the blue curve in Figure 2a, the transmission profile of the Ge waveguide showcases distinct Fabry–Perot resonances across the DFB-QCL laser’s wavelength range, which spans from 1286 cm^−1^ to 1290 cm^−1^. To determine the waveguide loss, denoted as *α*, we infer the losses from the fringe contrast ratio. The losses can be inferred using $\alpha =-\frac{1}{L}\ln\left(\frac{1}{R}\frac{\sqrt{\zeta }-1}{\sqrt{\zeta }+1}\right)$ [34], where *L* is the length of the Ge waveguide, being 4.5 mm, *R* is the facet reflectivity at the Ge/air interface, taken to be 0.36, and *ζ* represents the ratio between the maximum and minimum of the Fabry–Perot (FP) resonances, defined as $\frac{T_{\max}}{T_{\min}}$ .

As there was some nonuniformity in the resonance heights, we employed a correction technique to correct the FP resonances based on the local resonance peak. We adopted a piecewise cubic Hermite interpolating polynomial (PCHIP) to accurately fit the resonance peaks, as PCHIP fits preserve the original shape and monotonicity of the data. Once the PCHIP fit was made, the entire scan was normalized by dividing by this fit, resulting in corrected data that effectively scales the peak values to unity, as depicted by the red curve in Figure 2a. This approach ensures a robust depiction of the waveguide’s resonance behavior, pivotal for accurate waveguide loss computations. This yields an optical loss of approximately 1 cm^−1^ at 1289.6 cm^−1^, competitive with previous suspended Ge waveguides in the LWIR range [35]. For a more precise representation, we fit our corrected spectra to the transmission function of a Fabry–Perot, assuming a constant loss within the narrow spectral range of 1286 cm^−1^ to 1290 cm^−1^. The outcome of this fitting process, showcased by the purple curve in Figure 2a, suggested an average waveguide loss of approximately 1.2 cm^−1^ with a standard deviation of 0.2 cm^−1^ across the 4 cm^−1^ range.

It’s worth noting that our calculations utilized the reflection coefficient of an ideal end-facet Ge-air mirror. This method likely overestimates the waveguide losses since one of the end facets was created by dicing shown in the inset of Figure 1a. Unlike cleaving, dicing typically results in a rougher facet finish, reducing the reflection coefficient. As the waveguide’s performance is unaffected by end-facet quality, the reported waveguide losses here are a conservative estimate.

Finite element method (FEM) simulations were performed in order to confirm that the observed resonance stems from the waveguide cavity’s end-facet reflection. We have simulated the fundamental TE and TM modes of the waveguide. The effective refractive index of the TM mode (3.88) is slightly smaller than that of the TE mode (3.89). This is because the thickness of the waveguide is smaller than the width of the waveguide, and there is stronger confinement in the thickness direction of the waveguide along the electrical field of the fundamental TM mode. These modes are primarily confined within the Ge segment of the GOZ platform, as depicted in Figure 2b. This confinement is due to the substantial refractive index difference between Ge (*n* = 4) and ZnSe (*n* = 2.42 at 8 µm). Given the curved waveguide’s approximate length of 4.5 mm with an effective index of 3.89, the expected free spectral range of around 0.3 cm^−1^ matches well with the resonances observed in Figure 2a.

## 4 Thermo-optic tuning

To further assess the capabilities and resilience of the newly developed GOZ platform, we conducted thermo-optic tuning experiments on our Ge waveguide. The waveguide can serve as an optical phase modulator through thermal tuning, given the temperature-dependent variation in the material’s refractive index. Without adjusting the laser wavelength, peak or trough transmission can be achieved by operating the waveguide at on- or off-resonance temperatures, respectively. Temperature change induces a round-trip phase shift of $\frac{\partial \phi }{\partial T}=2L\frac{\omega }{c}\frac{\partial n}{\partial T}$ , where *ϕ* is the phase shift, *n* is the effective index, *L* is the length, and *ω* is the angular frequency. This results in a shift in the resonance frequencies of $\frac{\partial \nu }{\partial T}=-\frac{\nu }{n}\frac{\partial n}{\partial T}$ .

**Figure 3:**
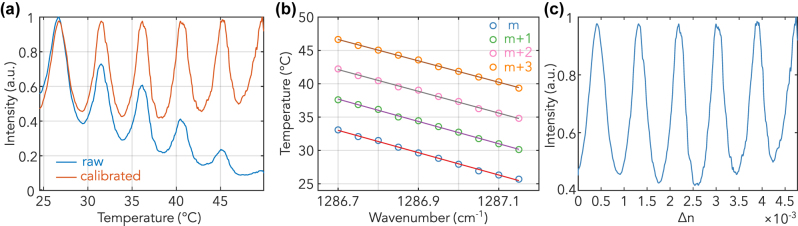
Characterization of waveguide loss through thermal tuning. (a) Thermal tuning of the waveguide to induce changes in its refractive index, with the raw data depicted in blue and the corrected data in red. Clear FP resonances are observed, indicating the effectiveness of the thermal tuning in characterizing waveguide loss. (b) Detailed characterization across wavenumbers from 1286.7 cm^−1^ to 1287.15 cm^−1^ with intervals of 0.05 cm^−1^. The varied colors represent distinct resonant peaks corresponding to different modes. (c) A representation of the results, where the *x*-axis has been transformed to display changes in the refractive index, further emphasizing the observed FP modes.

For our measurements, we incorporated a resistive heater consisting of two resistors and a thermistor with the GOZ platform to regulate its temperature. This integration was achieved by mounting the GOZ ZnSe chip on the front side of a silicon substrate using Arctic MX-4 thermal paste to provide heat transfer. The resistive heater and thermistor were mounted on the back side of the Si carrier chip in the same method. Additionally, a small amount of optical glue was applied at the sides of both the heater and thermistor. The temperature on the chip was monitored and controlled using a PID controller. Figure 3a displays the transmission spectrum from the thermal tuning experiment using the DFB-QCL laser at 1287.15 cm^−1^. As the GOZ chip’s temperature was adjusted from 25 °C to 50 °C, we observed five complete resonances. However, the absolute transmitted intensity decreased with increasing temperature, likely due to the measurement setup’s thermal expansion, especially due to thermal paste and wax, causing a shift in the waveguide’s end-facet position [34]. Consequently, the coupling efficiency diminished, leading to a decrease in transmitted intensity. After fitting the trend of both peaks and troughs, the corrected transmitted intensity is depicted as the orange line in Figure 3a. The resonance amplitudes below 40 °C align well with our laser tuning measurements from Figure 2a. An evident increase in the transmission troughs’ intensity was observed when the temperature exceeded 35 °C, possibly resulting from enhanced multiphonon absorption at elevated temperatures [36].

Next, we fine-tuned the DFB-QCL laser wavelength in increments of 0.05 cm^−1^ to carry out the thermo-optic tuning experiments. The in-phase resonant peaks, categorized by mode numbers, are illustrated in Figure 3b. To determine the correlation between wavenumber and temperature, we employed a linear fitting approach, applying a first-order polynomial to the data points. This analysis spanned wavenumbers from 1286.7 cm^−1^ to 1287.15 cm^−1^, providing insights into the thermo-optic characteristics of the waveguide. The observed groups consistently followed a linear trajectory, running parallel to one another, with a thermo-optic tuning rate of −0.06 cm^−1^ °C. Using the effective refractive index of 3.89 for the Ge waveguide, as derived from FEM simulations, and considering a waveguide length of approximately 4.5 mm, we deduced a thermo-optic coefficient $\frac{dn}{dT}$ of approximately 2 × 10^−4^ K^−1^. This value is smaller than the reported value of 4 × 10^−4^ K^−1^ in intrinsic Ge [37], a discrepancy that could possibly be explained by a difference between the measured temperature and the actual temperature of the ZnSe chip, since the heater and sensor were not exactly colocated. This value is also competitive when compared to the thermo-optic coefficients of other prominent materials, such as Si_3_N_4_ and SiO_2_ in the near-infrared telecom band around 1550 nm [38]. By correlating temperature tuning with changes in the refractive index, we can convert the temperature shift into a refractive index shift as shown in Figure 3c. This highlights the capabilities of the GOZ platform for constructing devices such as heater-based phase modulators.

## 5 Broadband loss measurements

Ge is renowned for its broad transparency window, spanning from 2 µm to 14 µm, encompassing both the mid-infrared and longwave infrared regions. To fully harness the advantages of Ge’s transparency window, it is imperative to complement it with a substrate or cladding material that matches its broad transparency range. In this context, ZnSe stands out as an ideal choice, boasting a transparency window that stretches from the visible spectrum to 16 µm. As a result, the GOZ platform naturally offers an expansive transparency window.

**Figure 4:**
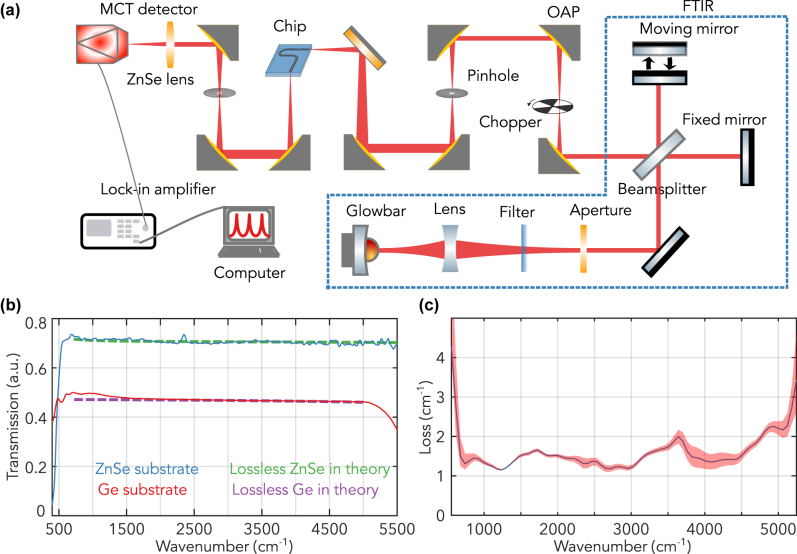
Broadband transmission assessment of the GOZ waveguide. (a) Diagram detailing the optical components and path utilized for the broadband transmission measurement. (b) Transmission window measurements for pristine Ge and ZnSe wafers, emphasizing the extensive transparency window of both materials. The theoretical lossless transmissions (no absorption and scattering loss) $\left(T=\frac{2n}{{\left(1+n\right)}^{2}}\right)$ for ZnSe [39] and Ge [40] are represented by green and purple dashed lines, respectively, and closely align with our measured transmissions, depicted by blue and red curves. (c) Detailed representation of the transparency window, showcasing the optical loss and transmission bandwidth of the Ge waveguide on the GOZ platform. The shaded region indicates one standard deviation.

To verify the transparency windows of Ge and ZnSe, we conducted detailed transmission measurements on pristine ZnSe and Ge wafers. The results, depicted in Figure 4b, demonstrate transmissions surpassing 70 % for ZnSe and 48 % for Ge. From the same figure, the theoretical lossless transmission (no absorption and scattering loss) of ZnSe (represented by the green dashed line) closely aligns with our measured transmission of our substrate ZnSe (depicted by the blue curve). Similarly, the theoretical lossless Ge (indicated by the purple dashed line) matches our measured transmission of our substrate Ge (shown by the red curve). These observations align with literature, which suggests ZnSe’s transparency window ranges from 0.5 µm to 16 µm and Ge’s from 2 µm to 14 µm [21], [31], further validating the high quality of the substrate’s transmission we utilized in our experiments.

To demonstrate the GOZ platform’s broad transparency window, we utilized the glowbar inside a Fourier Transform Infrared Spectrometer (FTIR) as the light source for transmission measurement through the Ge waveguide. The detail of the optical measurement setup is drawn in Figure 4a using an FTIR in step-scan mode. The infrared emission from the glowbar was first collimated, filtered, and modulated by FTIR, and then it passed a series of OAP mirrors to expand the beam and was focused on the end facet of the input side of the Ge waveguide. Broadband transmission measurements were then recorded using an MCT detector connected to a lock-in amplifier as shown in Figure 4a. The transmitted spectrum from the GOZ platform’s Ge waveguide is presented in Figure 4b, and the spectrum was calibrated using the Fabry–Perot measurements at 7.8 µm. A clear transmission plateau, spanning from 700 cm^−1^ to 5000 cm^−1^, corresponds to Ge’s 2 µm–14 µm transparency window, with an optical loss near 1 cm^−1^ over most of the range. Previous studies have demonstrated a few instances of low-loss and broadband waveguides in the LWIR range integrated on a platform. An example is the work by David et al. [25], which utilized a Ge slab on a gold layer. In comparison, the GOZ platform results in lower optical loss as it utilizes the nonmetallic ZnSe substrate, holding its application potential in broad transparency scenarios, particularly where Si-based materials like Ge on Si or SiGe on Si encounter limitations due to substantial multiphonon absorption beyond 7 µm. We note that there is no observable oxidation/degradation within approximately 1 month of experimental time. This could be due to a relatively thin layer of germanium oxide compared to the wavelength of the LWIR light [41]. This work validates the GOZ approach and underlines its capability for applications necessitating extensive transparency windows.

## 6 Conclusions

To our knowledge, this work represents the first demonstration of the low-loss GOZ photonic platform. We performed a systematic characterization of the Ge waveguide on GOZ platform using waveguide transmission measurements, achieved by both modulating the laser wavelength with the single-mode DFB-QCL and employing thermo-optical tuning by adjusting the temperature of the ZnSe chip. Both the laser tuning and thermo-optical tuning methods yielded consistent resonance results, aligning with an optical loss of 1.2 cm^−1^ at 7.8 µm under ambient conditions. Further examination of the GOZ Ge waveguide’s transparency window, using a broadband IR source in tandem with a FTIR, confirmed that the GOZ Ge waveguide retains similar transparency attributes as pure Ge. This finding demonstrates the potential of the GOZ platform in facilitating broadband IR and THz applications. Moreover, low-loss integrated photonic platforms play a critical role in facilitating on-chip nonlinear quantum processes, such as spontaneous four-wave mixing for entangled photon pair generation [42]. Presently, these processes are not observed in the LWIR spectrum, primarily due to the high optical losses of integrated photonic platforms. As optical losses in the LWIR are further minimized, these low-loss platforms will become instrumental for future quantum sensing beyond the classic limit.

Although our waveguides were not as low-loss as pure Ge, this is likely a consequence of sidewall roughness and the polycrystalline substrate. It is also conceivable that residual oxides in the Ge–ZnSe interface could play a role; future work will likely mitigate these effects, e.g., using chalcogenide glass as an upper cladding layer after the *in situ* removal of the native oxide of Ge. Our findings establish the GOZ platform as a versatile platform for LWIR integrated photonics, one that could eventually be as versatile as SOI has been at shorter wavelengths.
